# Case Report: Head injury presenting as lower cranial nerve palsy

**DOI:** 10.3389/fsurg.2025.1613887

**Published:** 2025-10-30

**Authors:** Shubham Gupta, Rakesh Gupta, Zafar Sheikh, Abhijeet Bele

**Affiliations:** Department of Neurosurgery, Mahatma Gandhi Memorial Medical College and Maharaja Yeshwantrao Hospital, Indore, Madhya Pradesh, India

**Keywords:** cranial nerve palsy, head injury, lower cranial nerve, occipital condyle, trauma

## Abstract

Lower cranial nerve palsy is a rare condition, most commonly caused by malignant skull base lesions or internal carotid artery dissection, and only rarely resulting from trauma. We report two cases of road traffic accident victims presenting with lower cranial nerve palsy involving cranial nerves IX, X, and XI. Both patients had similar clinical complaints. The most important findings were a dislocated occipital condyle fracture and suspected associated soft tissue injury. Both were treated conservatively with a rigid cervical collar. A nasogastric tube was required for the first patient during the initial recovery period due to severe dysphagia, while the second patient tolerated oral intake and did not require enteral feeding. Both patients showed gradual improvement in symptoms during hospitalization and follow-up. These injuries, with their often subtle clinical presentation, may be easily overlooked and require prompt detection to prevent complications.

## Introduction

Lower cranial nerve palsy is a rare condition, attributed to malignant skull base lesions such as multiple myeloma (1), metastasis of prostate cancer (2, 3), internal carotid dissection (4, 5), hypoglossal schwannoma (6), and Jefferson fracture (7, 8), among other causes. Symptoms can present slowly over time or suddenly (i.e., after trauma), depending on the cause, which affects the prognosis. Lower cranial nerve palsy is also associated with occipital condyle fractures (OCF) as described by Bell in 1817 (9). These injuries are rarely reported in the literature, and it is unclear whether these fractures are rare or underdiagnosed. They have been seen more frequently during the past decade, which may be due to the increased use of computed tomography (CT) and magnetic resonance imaging (MRI) scans (10, 11). These fractures are typically accompanied by other injuries of the cervical spine and are rarely seen as isolated injuries. One study found that 22% of patients with OCF had associated injuries of the cervical spine, with the majority of injuries being fractures of C1 and C2 vertebrae (12). In 1915, Collet became the first person to describe “glossolaryngoscapulopharyngeal” hemiplegia in a patient with a gunshot injury to the mastoid, which involved the four lower cranial nerves (13). Two years later, in 1917, Sicard described it as “The syndrome of the condyloposterior lacerated foramen” (14) with clinical presentations of hoarseness, difficulty swallowing (cranial nerves IX and X), and shoulder and neck weakness (cranial nerve XI), after which it came to be known as the Collet–Sicard Syndrome. We report two cases of lower cranial nerve palsy with occipital condyle fracture due to a road traffic accident (RTA). These injuries with subtle clinical symptoms may be overlooked and need prompt attention to avoid complications.

## Case report

**Figure 1:**
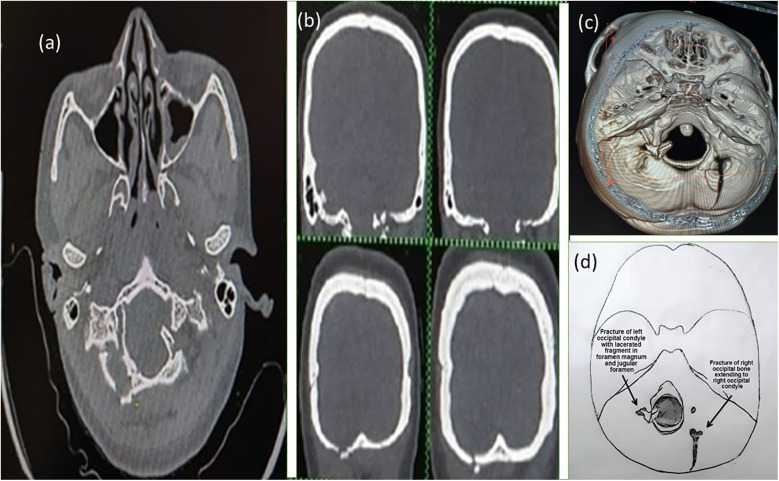
Axial **(a)** and coronal **(b)** section NCCT scan of the first patient with virtual reconstruction **(c)** and pictorial diagram **(d)** of the fracture (axial craniocaudal view).

**Figure 2:**
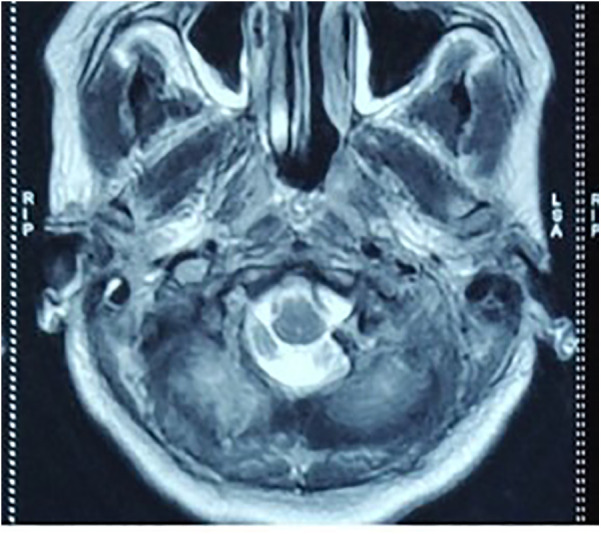
MRI of the first patient showing a lacerated fragment of the fractured occipital condyle in the foramen magnum without any neurovascular compression.

A 26-year-old woman presented to the emergency department following an RTA. At presentation, the Glasgow Coma Score (GCS) of the patient was E4V5M6, but she was clinically drowsy and easily roused with a normal bilateral pupillary response. She had no associated injury. On examination, the patient had normal motor and sensory responses in all four limbs. Local examination revealed contusions over the left mastoid region and the left lateral aspect of the neck. An non-contrast computed tomography (NCCT) brain scan was performed, which was suggestive of a fracture of the left occipital condyle with a lacerated fragment in the foramen magnum extending into the jugular foramen (Figures 1a–c). The right occipital bone was also fractured. The patient began to experience difficulty swallowing, accompanied by regurgitation of liquid from the nose. She showed signs of IX and X cranial nerve palsy. On examination, she also had left winging of the scapula, indicating XI cranial nerve palsy. MRI was advised, which showed mild pre-vertebral soft tissue thickening with no compression over any neural structure by a dislocated fragment or any ligament instability (Figure 2). She was managed conservatively and fitted with a hard cervical collar, and she remained hospitalized for 7 days. Over the 7 days, she was fed via a nasogastric tube, which was removed as her swallowing function improved. Gradually, her symptoms improved. At a follow-up examination 3 months after the injury, she had achieved complete recovery with minimal residual neck pain.

**Figure 3:**
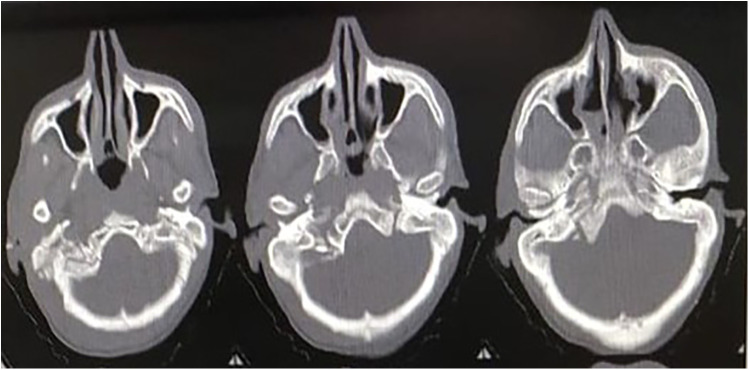
NCCT of the second patient showing a comminuted, undisplaced fracture of the right occipital condyle with a small chip in the jugular foramen.

Another patient, a 45-year-old man, following an RTA, presented to the hospital with GCS E4V5M6, without any other injury. The following day, the patient began to notice hoarseness of voice, difficulty swallowing, and limited movement in the right shoulder. An NCCT brain scan was done, which was suggestive of a fracture of the right mastoid bone with extension to the occipital bone and a comminuted, undisplaced fracture of the right occipital condyle with a small chip in the jugular foramen (Figure 3). He was managed conservatively and fitted with a hard cervical collar for 3 months. His swallowing difficulty was mild due to which nasogastric tube feeding was not required. He showed improvement in his symptoms and remained symptom-free after 3 months.

At the 3-month follow-up, both patients had complete resolution of lower cranial nerve deficits. The first patient reported only minimal residual neck pain, while the second patient remained entirely asymptomatic. Both patients actively participated in their care and recovery. The first patient reported that, initially, swallowing with oral intake was difficult and stressful, making nasogastric feeding uncomfortable but necessary. She appreciated the guidance and support from the neurosurgeon and noted a gradual improvement in her swallowing as the first week progressed. By 3 months, she expressed satisfaction with her recovery and minimal residual neck discomfort.

The second patient, with milder symptoms, reported only minor difficulty with swallowing and hoarseness, which did not significantly affect daily activities. He found the cervical collar manageable and experienced no significant discomfort or limitation during the treatment period. Both patients underscored the importance of careful monitoring by and communication with the treating team, which helped them cope with the rare and complex nature of their injuries.

## Discussion

The clinical symptoms shown by both patients were cranial nerve palsy of cranial nerves IX, X, and XI. Neither of the patients had weakness in any limb or dysfunction of the bowel or bladder. Occipital condyle fractures are generally caused by high-energy blunt trauma. In most cases, neurological deficits are attributed more to the severity of the head and neck injury than to the occipital condyle fracture itself. Such patients generally have polytrauma and do not survive the initial trauma (15). Both patients developed paralysis of the sternocleidomastoid and trapezius muscles and hoarseness in addition to difficulty swallowing with a deviation of the palate, indicating palsy of cranial nerves IX, X, and XI. The most important findings in our case report were a dislocated occipital condyle fracture and suspected soft tissue injury. Alar ligament thickening, stretching, or discontinuity is a useful, direct CT sign of disruption, but we cannot confidently diagnose the integrity of the alar ligaments using these criteria because of the frequent presence of minor positional asymmetry (16). Anderson and Montesano (17) published a classification guideline in 1988 based on a retrospective review of six patients who were scanned using either CT or conventional tomography. Tuli et al. modified the above guidelines in 1997 (18), determining instability according to CT and MRI scans, considering the extent of ligamentous injury and occiput-C1-C2 rotation and translation. Tuli et al. divided the classification into three types, based on dislocation, rotation, and translation of the O-C1-C2 complex and ligamentous injury verified by MRI (Table 1).

**Table 1:** Tuli's classification of occipital condyle fracture.

Type	Description	Stability
Type 1	Undisplaced fracture	Stable
Type 2A	A displaced fracture with no findings of ligamentous injury is demonstrated on MRI, and without rotation or translation in the O-C1-C2 complex	Stable
Type 2B	A displaced fracture of the occipital condyle with MRI evidence of ligamentous disruption or rotation and translation in the CT scan	Unstable

The first patient had a type 2A fracture, and the second patient had a type 1 fracture. The palsy of cranial nerves IX, X, and XI was likely already present directly after the accident, which was noticed the next day in both patients after starting the oral diet. Hanson et al. supported the hypothesis that disruption of all craniocervical ligaments is required for complete occipitoatlantal dissociation, and stabilization of a unilateral condyle avulsion fracture is maintained by an intact contralateral alar ligament and tectorial membrane (16). Both of our patients were RTA victims, consistent with prior studies identifying RTAs as the most frequent cause of cranial nerve injuries (19, 20). While Basheer et al. and Swamiyappan et al. reported facial nerve (CN VII) palsy as the most common injury, Coello et al. identified the olfactory nerve (CN I) as the most common cranial nerve involved in head injury patients; however, all three studies noted that lower cranial nerve (IX–XII) involvement was rare (19–21). In contrast, our patients developed isolated lower cranial nerve deficits (IX–XI) following occipital condyle fractures, with no involvement of the more commonly affected nerves. The likely biomechanical mechanism in our patients was a combination of axial loading and lateral bending, producing occipital condyle fractures with fracture fragments extending toward the jugular foramen. However, the exact mechanism is still unclear. This close anatomical relationship predisposes cranial nerves IX, X, and XI to stretching, avulsion, or compression. The absence of persistent compression on MRI, along with eventual complete recovery, suggests that transient nerve stretch or traction was the most plausible mechanism, consistent with prior reports of lower cranial neuropathy in occipital condyle fractures.

Immobilization with a hard collar is recommended in stable OCF without bone fragment displacement because it could migrate with head movements (22). There is no sufficient evidence showing that patients treated with a hard neck collar have a better outcome than patients who do not receive any treatment. Most studies recommend treatment of occipital condyle fractures with a hard neck collar or a halo frame for 6–12 weeks. Tuli et al. (18) recommended that undisplaced fractures without ligamentous injury (type 1) not be immobilized, but that type 2A fractures should be treated with a hard collar. Type 2B should be treated with a halo vest or by surgical fixation. There are reports of three patients (12) with potentially unstable fractures who were operated on to remove the dislocated fragment. In these cases, all patients showed improvement in cranial nerve palsy; however, it is unclear whether the surgery was responsible for the recovery of nerve function. The strength of our study lies in the detailed clinical documentation, radiological correlation, and adherence to conservative management protocols with favorable outcomes. However, limitations must be acknowledged. With only two cases, the findings cannot be generalized to all patients with occipital condyle fractures. Furthermore, while CT and MRI scans demonstrated fracture configuration and ruled out gross instability, subtle ligamentous or axonal injuries could not be confirmed, limiting the certainty of the proposed mechanisms of cranial nerve palsy. The follow-up period of 3 months, though adequate to document recovery, does not exclude the possibility of delayed instability or recurrence. Despite these limitations, our cases add to the sparse literature describing Collet–Sicard syndrome following occipital condyle fractures, particularly involving cranial nerves IX–XI, which is less frequently reported than XII nerve palsy.

## Conclusion

Lower cranial nerve palsy following occipital condyle fracture is a rare but clinically important manifestation of craniocervical trauma. These two cases highlight the potential for IX, X, and XI cranial nerve involvement due to the proximity of the jugular foramen to the occipital condyle—an association less commonly reported than hypoglossal nerve involvement. Although direct compression by the dislocated fragment cannot be excluded, axonal injury from traction or nerve root avulsion is the more likely mechanism, with onset probably occurring immediately after the accident. This study contributes to the limited literature on Collet–Sicard syndrome secondary to occipital condyle fractures and underscores the importance of prompt cranial nerve examination along with targeted CT and MRI of the craniocervical junction. Early recognition enables appropriate conservative management and can lead to favorable neurological recovery.

## Data Availability

The raw data supporting the conclusions of this article will be made available by the authors, without undue reservation.
